# Hospital and Institutional News

**Published:** 1916-01-08

**Authors:** 


### HOSPITAL AND INSTITUTIONAL NEWS.

### THE NEW YEAR'S HONOURS.

Not many of the recipients of New Year honours have had close association with hospitals, except some of the medical men who are in the list. Mr. W. W. Astor has subscribed munificently to the Red Cross and other special funds: he now becomes a Peer. The Hon. C. Russell, on whom a Privy Councillorship is bestowed, is chairman of the collections committee of the *Times* Red Cross Fund; and Sir G. Bullough, who receives a similar honour, turned his yacht into a hospital ship during the South African War. Mr. F. Gorell Barnes, D.L., J.P., who becomes a Knight, is chairman of the Greyhound Convalescent Home for wounded soldiers; and Mr. T. J. Hughes, who receives the same honour, is chairman of the National Health Insurance Commission for Wales. Mr. J. Howard, now knighted, has done much for hospitals in Brighton, and has recently endowed with £30,000 a memorial to Miss Cavell, consisting of twenty-four cottage homes for incapacitated nurses. Mr. J. G. Griffiths, who becomes a M.V.O., is the honorary secretary of King Edward's Hospital Fund for London. There are three medical Knights Bachelors: Mr. G. A. Berry, the well-known Edinburgh ophthalmic surgeon and an ex-President of the Edinburgh College of Surgeons; Mr. Milsom Rees, a London laryngologist, holding Court appointments; and Dr. T. W. Parkinson, a general practitioner of many friends and well-deserved reputation. Neither of the two last named holds any appointment at a pre-war voluntary hospital. The Colonial Office List is a short one, and contains no medical name but that of Surgeon-General G. C. Jones, of the Canadian Expeditionary Force, on whom the C.M.G. is conferred. In the Indian List we note that Dr. J. A. Turner, Executive Health Officer at Bombay, and Lieutenant-Colonel R. C. MacWatt, I.M.S., receive the C.I.E. There are eleven awards of the Kaisar-i-Hind Gold Medal, but none of them goes to a medical man.

### NAVY AND ARMY HONOURS.

Surgeon-General Sir A. Bowlby is appointed K.C.V.O.; and Staff-Surgeon R. J. Willan, R.N.V.R., a member of the same Order. It is hardly necessary to mention that the former is Surgeon-in-Ordinary to the King, and surgeon to St. Bartholomew's, and is already a K.C.M.G.; Mr. Willan is assistant surgeon to the Royal Victoria Infirmary, Newcastle-on-Tyne. Surgeons-General Sir James Porter, R.N., and Sir W. Watson Cheyne, Bart., are each appointed to be K.C.M.G.; the former was Director-General of the Navy Medical Service, but had retired when the war broke out; the latter is too well known as President of the Royal College of Surgeons and in a dozen other capacities to need further description —as a small point of interest it is noteworthy that his vigorous attack on some of the high officers of the Army Medical Service has not stood in the way of recognition of his services rendered to the Navy authorities. Five medical men become Companions of the Bath: they are Fleet-Surgeon A. Gaskell, R.N.; Surgeon-General W. H. Norman, R.N.; Brevet-Colonel W. W. White, I.M.S.; Surgeon-General H. D. Rolleston, R.N., senior physician to St. George's Hospital; and Dr. A. W. J. MacFadden, of the Local Government Board. Both the two last named served as civil surgeons in the South African War.

### NORWICH SECRETARYSHIP AGAIN VACANT.

Referring to our Note on the Norwich vacancy in The Hospital of October 23 last (p. 63), we have to report that the Board of the Norfolk and Norwich Hospital are re-advertising the appointment of secretary. The committee have now recognised the mistake they made in offering inadequate remuneration. From the advertisement which will be found in another column, it will be seen that the salary has been increased to £350, with board and residence in the hospital, for a single man, or to £450, without board and residence, for a married secretary. "Candidates must be ineligible for military "service, and must have had some administrative "experience." Even now, having regard to the importance of the Norfolk and Norwich Hospital as it exists to-day, the terms offered are not up to the responsibilities attaching to the vacant office if it is filled by an administrator of the first rank whose practical knowledge and experience of the duties to be fulfilled are adequate to their efficient performance. The opening, apart from the emoluments, is exceptionally good for a man of enterprise and capacity, considerations which may produce a competent candidate with the courage to rely upon the generosity of the hospital authorities when he proves a demonstrable success.

### THE DEFENDER OF CHITRAL.

The late Sir George Scott Robertson, M.P., who died on January 3, was better known to the public as the defender of Chitral and as a politician than as a medical man; nevertheless it was through his medical qualifications that Sir George got his chance in life. Born in London in 1852 of Scottish parents, he was educated at Westminster Hospital, whence he qualified as L.S.A. in 1876 and M.R.C.S. in 1877, and joined the Indian Medical Service in the following year, just in time for the Afghan War of 1879-80, including the siege of Sherpur. He then spent eight years in the Central Provinces before being transferred to Gilgit on the North-West Frontier. Here his diplomatic gifts were speedily recognised, and he became a Political Agent for dealing with the wild tribesmen; probably his medical knowledge stood him in good stead in gaining their confidence. After various confidential and important missions he was besieged in 1895 in Chitral with a garrison of but 543 Sikhs. Though seriously wounded he held out for seven weeks until relieved, and was made a K.C.S.I. for his services. Returning home in 1899, he entered politics, and after a defeat in 1900, was elected for Bradford in the Liberal interest in 1906. He was twice married, and leaves a widow and one daughter.

### MR. CARNT'S DEPARTURE FROM MANCHESTER.

The year's work at Manchester Royal Infirmary was not allowed to close without a final official reference to the work of Mr. W. G. Carnt, who, as our readers will remember, lately retired from the post of general superintendent. At a meeting of the board on December 29 it was stated that Mr. Carnt had left the institution, and that Mr. F. G. Hazell, his successor, had entered upon his new duties. Reference was made to the house committee's resolution of sympathy with Mr. Carnt in the illness which occasioned his resignation, and the committee expressed their " admiration of his unsurpassed knowledge of hospital construction and administration," together with their hope that he would benefit by removal to a milder climate. In this hope our readers will share, and Mr. Carnt's departure, thus recorded at the end of the year, will be read of sympathetically by all who have been acquainted with his work, or known how regretfully he has been compelled to limit his activities in it. Another loss experienced by the staff of the Royal Infirmary lately occurred through the death of Sister French, whose connection with the nursing staff had lasted for thirty-three years.

### THE ATTENDANCE AT ANNUAL MEETINGS.

Is it a sign of an institution's health when there is only a small attendance of the governors at the annual meeting? Recently a governor at the annual meeting of a well-known hospital rose and remarked that it must be rather disappointing to the chairman and the board of management to see so few subscribers present. In replying to this remark, the chairman admitted that he and his colleagues would, of course, like to see a larger number of subscribers, but he said he might also be forgiven if he looked upon a small attendance as a proof that the great bulk of the subscribers were so thoroughly satisfied with the way in which the institution was managed, that they were content to leave matters in the hands of the board and the officials. At the present time there is no doubt that many hospital subscribers are themselves engaged in active work connected with the war. Doubtless, this fact contributes largely to the small attendances which have been seen of late at many annual meetings of institutions.

### RED CROSS HOSPITALS AND SUNDAY CINEMATOGRAPH SHOWS.

In May 1912 some controversy was aroused over the question of hospitals benefiting from Sunday cinematograph shows. The immediate advantage to those institutions which agreed to accept the profits resulting from the Sunday takings was obvious, but wherever contributions from such shows were accepted, the withdrawal of Church support was threatened, and this entailed the possibility of disaster to the Hospital Sunday Fund movement. Our view was that individual hospitals must be sufficiently public-spirited to look beyond their own and immediate advantage, and, without reference to the rights and wrongs of the dispute, abstain from accepting any contribution which might involve injury to the voluntary system, dependent as this largely is on Church support. These facts are only worth reviving now because at the opening of a new Red Cross Hospital just before Christmas it was stated that the only regular income on which the new institution could rely was the profits of the Sunday lettings of the local cinematograph theatre. It was added, however, that the theatre would be open in any case, and that " as the proceeds have to be devoted to a charitable cause," the hospital was at least as deserving as any other. In this case no objection seems so far to have been raised, but at the same time the situation has not changed, and it may be well, therefore, to remind the new institution of the difficulties which had to be faced by the hospitals that accepted contributions from this source a year or two ago.

### ON CUTTING DOWN THE ANNUAL REPORT.

In response to the demand for rigid economy, some of the county medical officers have been cutting down the printing bill of their annual reports by omitting certain normal features in them. A similar course has also been considered by certain hospital authorities; and the question arises as to the wisdom of such a retrenchment. The annual reports of the county medical officers are sometimes of ample proportions, but in the case of hospitals they vary from small pamphlets to a hundred small pages or so. As an instance of the reduction which is now being made or suggested, we may record the decision of one hospital to omit from its annual report the list of the different diseases for which the patients have been treated. No up-to-date hospital of the modern type which aims at efficiency and ample public support will be foolish enough to sanction the evisceration of its annual report. The present time enables these reports to be made full of interest for everybody. The record of facts, officials, and statistics must be presented, but the manner of presentation varies from a bald and repulsive summary to a carefully edited and illustrated survey of the institution's life and work. The former type is unworthy, but the latter may prove a gold-mine in the results it brings to the Treasury.

### CEREBRO-SPINAL MENINGITIS.

So far but little has been heard of this disease in England this winter, in spite of some dismal prophecies which were bandied about last summer. Whether this absence of news is due to the censorship, or to fear of it, or whether it means that no epidemic has occurred remains to be seen. In Australia the recent serious outbreak has been quenched in every province except Victoria, where the figures are still distinctly unsatisfactory and the authorities are being accused of apathy. It seems that the use of anti-meningococcus serum obtained from America has met with no success, and it has been decided to set up a laboratory for the manufacture of sera in Australia itself; advertisements have already appeared in this country for a duly qualified expert to take charge of the projected institution. Opinion on the value of Flexner's serum is still divided in this country, with a preponderance (numerically) against it. In the January number of the *Practitioner* several doctors who used it extensively in this country last year record their impressions: all of them pronounce it useless except one, and he (Dr. A. G. Robb) admits that his results were less favourable last year than they had been in some previous epidemics when he had employed it. Simple lumbar puncture, repeated regularly and frequently, finds a good deal of favour, and emphasis is laid on the importance of administering a general anæsthetic for the process, in spite of a risk which is said, contrary to some published opinions, to be practically negligible.

### AN ENCOURAGING EXAMPLE.

FROM the hospital point of view the New Year has begun well and hopefully. After seventeen months of war we have seen what really amounts to a revival of Hospital Sunday, to the raising of a third million for the Red Cross funds, and in general to the proof that the voluntary system has gained strength from the war, notwithstanding the practical difficulties of high prices and a shortage of staff. This note of hopefulness and strength, which is very welcome at the beginning of another year, was happily struck by Mr. H. H. Bedford, the chairman of Sheffield Royal Infirmary, at the recent quarterly meeting, when he remarked that the response to the special appeal had been very generous, and that the honorary staff had nobly risen to the occasion and, by undertaking the extra work that resulted from a shortage of resident medical officers, had enabled the work to go on as well as, if not better than, had been expected. Since a few institutions are heard complaining that they cannot raise the money which they need, we are glad to draw attention to the success of the special appeal at Sheffield, as it bears out our contention that for a properly managed institution the present is a good time for rallying public support. Appeals in such days as these have to be issued for suitable objects, but where the need is real and the institution well administered the response is not far to seek.

### "THE BOYS IN BLUE."

A GRACEFUL tribute to the enterprise and good will of those who organised the Christmas festivities for hospital patients, which we described last week, is contained in the "note of recognition sent by the wounded soldiers at Addenbrooke's Hospital to the secretary-superintendent, Mr. R. J. Coles, signed by "the boys in blue" in the Griffith, Albert, and Bowtell wards. The note states that "on behalf of our fellow-comrades we tender our united appreciation to all concerned for catering, comfort, and entertainment given on our behalf during Christmastide." The writers "especially wish to thank the committee, the secretary-superintendent, and matron for permission granted us in order that such entertainment could be carried through." They add that the principal attraction was "Addenbrooke's Own Troupe." No better thanks or praise could be desired by any than is contained in this last sentence; and there can be little doubt that the above note of recognition is the type of many others inspired by the efforts of hospital staffs and other helpers to give the patients a happy Christmas Day. It forms a charming postscript to the account which appeared in our columns.

### HOW BATH IS TACKLING THE MEASLES PROBLEM.

LOCAL authorities are stirring themselves to varying degrees of activity to meet the requirements of the recent Local Government Order relating to the notification of measles and provision for nursing assistance in suitable cases. The latter item will necessarily mean an increased expenditure, which at the present time committees are likely to sanction with reluctance. It may, however, be confidently expected that the ventilation of the subject will impress upon the public the seriousness of the loss of young lives that is attributable directly or indirectly to epidemics of measles. In the city of Bath, the medical officer of health, Dr. W. H. Symons, has represented to his committee the danger of delay in dealing with this public health question; he stated that an epidemic of measles was now due in Bath in accordance with observations which led him to expect such an epidemic every three years with very fair regularity. His recommendations include provision for nursing cases in their homes, and for treatment in hospital of a certain number of cases. At least twenty cots are considered necessary for children alone, and if it be deemed advisable to provide for older persons, another ten beds will be wanted. Dr. Symons suggested that it would be easy to fit up a house in a central district, and gave estimates of the cost. The whole matter is still under the consideration of the hospital committee, but it seems likely that Bath will not lag behind in dealing with the measles danger.

### THE TRYING TIME OF THE SURGICAL AID SOCIETY.

FEW agencies within the last fifty years have done more to mitigate the woes of humanity than the Surgical Aid Society, whilst at a period of war the calls upon its bounty increase. It is, therefore, all the more regrettable to learn that the funds have fallen off. There are very few of the original founders of the society in 1862 now alive, except Sir Thomas Crosby, M.D.; Mr. Samuel Watson, the present treasurer, has been a supporter since 1869, and his father was amongst the founders. With the war have come fresh and additional calls for assistance, not the least being from the sailors and soldiers of Belgium, whose wants have been supplied with alacrity. Last year alone the society distributed about 4,000 trusses, 2,000 abdominal belts, 4,000 special boots, and 1,000 legs and arms; artificial teeth have also been provided for men who otherwise would have been ineligible for the Army. But at the same time the annual subscriptions have decreased by £1,400, the life subscriptions by £1,220, and the legacies by £7,600. Moreover, there has been a decrease in sums received from patients, who hitherto have contributed the whole or part of the cost of the instruments they have required. There has been a general cutting down in managerial expenses, but it is doubtful if this will cover the loss due to the war. It would be a great pity if the work of such a useful society had to be curtailed through inadequate financial support, and it is trusted that in the coming year the society will recover the losses it has sustained.

### THE NORTHERN MINERS' AMBULANCES.

On December 23 the King inspected in the grounds of Buckingham Palace twenty-five fully-equipped motor-ambulances, provided for foreign service by the miners of Lancashire and Cheshire, and presented by them to the Red Cross. Another twenty-five cars are to be presented shortly by the colliery owners of the same counties. The ambulances are specially designed for heavy work, and were closely inspected by the King: one car as a type was examined in detail. The King expressed his approval of the completeness of the equipment, and also his keen appreciation of the generosity of the miners. Among those present were Lord Derby and his brother, the Hon. Arthur Stanley (Chairman of the British Red Cross Society); Sir John Thursby, Bart., Chairman of the Lancashire and Cheshire Coal Association; Mr. Walsh, M.P. for Ince, Lancashire; and Mr. Greenall, President of the Lancashire and Cheshire Miners' Federation.

### THE ORDER OF ST. JOHN.

On the nomination of the Sub-Prior, Lord Plymouth, and with the approval of the Grand Prior, the Duke of Connaught, Mr. Evelyn Cecil, M.P., has been appointed Secretary-General of the Order of St. John of Jerusalem in England, in succession to the late Rt. Hon. Sir Claude Macdonald. Mr. Cecil is already a Knight of Grace of the Order; he was assistant private secretary to his uncle, the Marquis of Salisbury, when Prime Minister in 1891-92 and 1895-1902, and was for five years a member of the old London School Board. His wife, a daughter of the first Lord Amherst of Hackney, is a great authority on gardening, and is a Lady of Justice of the Order of St. John. Mr. Cecil has been a member of Parliament since 1898.

### WASTAGE AMONG AUSTRO-GERMAN DOCTORS.

Some curious and very possibly "cooked" figures have been placed before the Hungarian Parliament concerning the wastage among the medical officers of the Central Empires during the war. As reported in the *Morning Post*, it was stated that up to November 1 last, 96 German and 101 Austrian doctors had been killed in action; 393 and 315 respectively had been wounded; 215 and 331 taken prisoners; 707 and 971 died of infectious disease (chiefly cholera and typhus). It seems incredible that such small numbers have been killed, unless the Austro-German practice is to have no doctors at all within two or three miles of the fire-trenches. On the other hand, the numbers who have died of disease are enormous; and if these figures are correct, it is obvious that terrific epidemics must have swept the armies of those Powers. We prefer to believe that for some reason, possibly connected with internal politics or possibly for consumption in neutral or Allied countries, the real figures have been concealed; and that very probably a large number of those alleged to have died of disease have in reality been killed in action.

### DUNDEE'S NEW HOSPITAL FOR INFANTS.

As was anticipated in a recent issue, the scheme to provide Dundee with a hospital for children under five years of age is bearing fruit. A house in Windsor Street, with accommodation for some twenty beds, is being prepared, and at an early meeting of the supporters of the scheme a constitution will be drafted, and the committee of management appointed. As already stated, it has been estimated that the sum of £2,000 would start the new institution and enable it to carry on its work in supplementing the existing baby clinics for three years. This sum, it is understood, has now been raised, and, therefore, while Dundee will still be without a children's hospital in the full sense of the word, its arrangements for "child welfare" will include some provision for institutional treatment without which such arrangements lose half their value. It is satisfactory to know that the new proposal has been promptly carried out, and that public opinion in Dundee is alive to the importance of making its system of baby clinics as complete as possible.

### EXTRA WORK AND ITS REWARD IN HOSPITAL OFFICES.

So much extra work has been thrown upon the secretarial departments during the past year that it is satisfactory to note the instances in which hospital committees have recognised this by giving additional remuneration to members of the office staff. At Portsmouth the committee of the Royal Hospital has given a gratuity of £5 to Mr. Shotter, the secretary's assistant, on account of the extra work that he has performed during the past four months, through the absence of one of the clerks on active service. Several members of the committee commended the manner in which this extra work had been carried out, and in view of the fact that in most cases the remuneration of the administrative staffs is in need of being raised, any testimony of appreciation from committees at the present time is to be welcomed. Extra work during the war, and its recognition, should prove an earnest of a general reconsideration of secretarial salaries after the war, for which public opinion will be ripe, thanks to the services the hospitals have rendered.

### A BRITISH AMBULANCE IN ITALY.

For some months past a party of British volunteer workers have been assisting in the convoy work of the Italian Army against the Austrians; and it is with much pleasure that we learn of the recent award of the silver medal "for military valour" to Mr. G. M. Trevelyan by the King of Italy. Mr. Trevelyan is a historian and a son of Sir G. O. Trevelyan, and is Commandant of the First British Ambulance Unit for Italy. The grant of the medal is made in flattering terms, and it is specifically stated that he partly represents his colleagues, whose conduct appears to have been equally gallant. The special circumstances in which the medal was gained were connected with the evacuation of a hospital in the village of San Florian, near the Gorizia front. The hospital was being heavily bombarded by the Austrians, and several of the wounded patients were killed during the process of removing them, as was also the Italian officer in charge of the hospital. Only two days previously a similar evacuation under heavy fire was carried out at a cholera hospital, on which occasion Mr. Trevelyan's car was riddled with shrapnel.

### BRITISH HOSPITALS FOR FRENCH WOUNDED.

We take from the *Gaulois* a list of the British voluntary organisations which have been established in France for the care of the French wounded. On a recent occasion we suggested that these works of mercy, greatly as they are appreciated by our Allies, absorb a large number of British doctors whose services might be more economically used and who may in the not distant future be required for our own Armies. Without emphasising this point again, upon which there is admittedly room for legitimate difference of opinion, the list is certainly even longer than we had suspected; no indication is given of the total number of beds administered, nor of the number of British medical men and nurses employed. There are twenty-seven such hospitals in all, and as this is the only enumeration of them which we have seen in this country, it may be worth while to give the whole list for future reference. Of some a good deal has been heard in the press lately; notably the Urgency Cases Hospital at Bar-le-Duc and the Arc-en-Barrois Hospitals. Many of the rest are evidently conducted by private munificence and are naturally less under the public gaze.

### THE "GAULOIS'" LIST.

Besides those just mentioned the Parisian newspaper enumerates the following:—The Hôpital Herbert Ward at Rolleboise; the Hôpital Lady Wemyss at Canly; the British Ambulance Committee at Gérardmer; the Hôpital Franco-Anglais (Lady Murray) at Tréport; the Hôpital FitzGerald at Passy; the Trianon Hospital at Versailles; the Hôpital de l'Alliance at Yvetot; the Hôpital Anglo-Ethiopien at Frévent; the Hôpital du Château de Béarn established at St. Cloud by Lady Hartwell; the Allhusen Hospital at Ceret; the Hôpital du Château d'Anuel near Compiègne; the Joint Anglo-French Hospital and Hôpital Civil de la Clayette; the Florence Fiennes Hospital at Malo-les-Bains; the Scottish Women's Hospital at Chanteloup; the Johnstone-Reckett Hospital at Ris-Orangis; the Hôpital du Château de Rozières near Nanteuil-le Haudouin; the Hôpital du Château de Blancat at Pau; the First Aid Nursing Yeomanry Hospital at Calais; the Lady Sykes Hospital at Canly; the Hôpital d'Hardelot near Boulogne, founded by the Duchess of Rutland; the Surgical Ambulance at Rosebruge (Mrs. Borden-Turner); the Hôpital Canadien No. 4 at St. Cloud; the Hôpital Anglais No. 307 at Neuilly-sur-Seine; the Hôpital Irlandais at Cezaincourt; the Hôpital Anglais No. 6 at Nevers. Truly a wonderful list.

### THE PERTH PUBLIC HOSPITAL, WESTERN AUSTRALIA.

We are always glad to receive reports from the hospitals of the Dominions, since they generally provide many interesting points to us at home, particularly in regard to the system of State grants which prevails in the Dependencies. In the case of the Perth Public Hospital, Western Australia, the report from which has now reached us, a notable point concerns the feeling in regard to the financial policy of the institution, for we learn that the continued efforts to attract donations and annual subscriptions have "not met with the success they deserve; the general opinion being that the Perth Hospital should be a State-supported hospital." As things are, the Government grant amounts to £19,180, fees from the patients total £3,326, while contributions amount only to £433. This gives some idea of the relative proportions in which the income is forthcoming. On the other hand, the work of the hospital is many-sided. Apart from dealing with some 3,000 in-patients, and similar numbers of out-patients and of casualties, there is an infectious diseases branch where 530 in-patients were received. Here there are sixty-six beds for consumptives and sixty-two beds for cases classified under the head of infectious diseases. The hospital possesses a ward with five beds for male venereal cases, and a consideration of the report of the chief resident medical officer shows that every department tends to develop, and when the varied nature of the cases treated is considered, the problems of administration which form a necessary part of the Perth Public Hospital's work are full of suggestion to hospital men in this country, where the broad division into general, special, and fever hospitals still holds good.

### ANTHRAX AND THE SHAVING-BRUSH.

Thanks to the industry and pertinacity of Mr. R. R. Elworthy, three mysterious cases of anthrax have been cleared up, and a hitherto unsuspected source of anthrax contagion has been laid bare. Last summer a man died in the West London Hospital after an illness of two and a half days. His symptoms were those of an intensely rapid cellulitis of the neck, originating in a small pustule, and after death it was demonstrated that they had been caused by the bacillus of anthrax. It was suggested at the inquest, but pooh-poohed in certain quarters, that the infection might have been inoculated while shaving from an infected brush. Inquiry revealed that the deceased had used a new shaving-brush, which, fortunately, had not been washed after use. To cut the story short, this brush was proved to harbour anthrax bacilli; and when five other brushes out of the same batch were procured from the shop, four of them were found to be inhabited by the same organism.

### NON-PANEL DOCTORS AND INSURED PATIENTS.

At a meeting of the Manchester Insurance Committee on December 28, the Chairman, Mr. Walter Davies, announced that, as a result of a conference between the Insurance and the Medical War Committees with the Panel Committee, a joint agreement had been arrived at whereby every insured person would be provided with an adequate medical service. He added that it had been impressed on non-panel doctors if medical men on the panel were released to go to the Front, it would be necessary for non-panel doctors to come to the assistance of insured persons. After the above conference Mr. Davies declared that a scheme had been decided on whereby practically all the Manchester doctors, on and off the panel, had agreed to attend the insured. The completeness of this mutual agreement has, however, been questioned, and it seems as if the scheme had been accepted in principle rather than in detail by non-panel practitioners of the city. The difficulty to be met is a real one, and while non-panel men may generally approve of the safeguarding of the practices of those doctors who are now absent, that is not quite the same thing as, for instance, consenting to put themselves under the control of Insurance Committees.

### ANTI-TYPHOID INOCULATION.

The latest available official figures concerning the incidence of typhoid fever in our troops in France, inoculated and non-inoculated, are those published by Mr. A. Fleming, F.R.C.S., of St. Mary's Hospital. Up to September 19, 1915, there were in our Expeditionary Force 1,052 cases of typhoid fever, with 146 deaths. Of these men 494 had been inoculated, 558 had not: incidentally it may be remarked that one of the speakers at a Royal Society of Medicine symposium in November was understood (and was reported) to say that there had been 494 cases all told, an obvious error but one which went uncorrected at the time. Among the 494 inoculated men there were 33 deaths; among the 558 uninoculated there were 113 deaths. In comparing these figures it is essential to know the (average) proportion of inoculated and uninoculated men in the Force. This it is practically impossible to get accurately, but it is reckoned that from May onwards over 90 per cent. were inoculated: in some divisions at least this percentage was reached as far back as last January, to our knowledge, and we should have thought that by the summer the percentage would have been not less than 95. However, taking the lesser figure of 90 per cent. inoculated, it appears that from May onwards there were 195 cases among these men, with thirteen deaths; and 77 cases among the uninoculated, with 13 deaths, that is to say, the uninoculated soldiers had a death-rate just nine times as high as the inoculated.

### SISTERS AS ANÆSTHETISTS.

The employment of trained nurses as anæsthetists in hospitals in this country is likely to give rise to controversy, as was suggested in these Notes last week. The point has been broached more than once in The Hospital, and reference has been made to foreign hospitals where the practice is in vogue, for example, in the Stockholm hospitals (see The Hospital for February 6, 1915, p. 426). The chief point in favour of allowing experienced sisters (no one of lower rank in the nursing world has been suggested for the task) to perform the duties of anæsthetists is their superiority as administrators over medical men who are not specialists in this practice. The young resident medical officer or the general practitioner who acts only intermittently as anæsthetist, and whose mind is occupied during the day by many other problems, is probably not a perfect administrator. Those who prefer a well-taught sister exclusively retained for this office are said to be justified in their preference by the results obtained, but the principle is one on which difference of opinion is bound to exist. No one has suggested that nurses, however conscientious and efficient, should be employed when the services of a medical man practising solely as an anæsthetist are obtainable. At the present time such men are scarcer than ever before, and the example of the Norfolk and Norwich Hospital may possibly find imitators, at any rate during the war.

### THIS WEEK'S DRUG MARKET.

Business has already revived, and will be in full swing again very shortly. The most noteworthy change is a considerable rise in the price of rectified spirit. This advance, which was anticipated, is due to a variety of causes, the main ones being the requirements of the Munitions Department, a shortage of raw material, and the heavy reduction of supplies from abroad. As a consequence of this advance, quotations have been raised for sweet spirit of nitre, aromatic spirit of ammonia, tinctures, and in fact of all preparations in the manufacture of which spirit is used. The higher prices will make a sensible difference to the drug bills of hospitals. Methylated spirit is in extremely short supply. A further advance in the value of glycerine is not unexpected. Castor oil continues to become scarcer and prices are advancing. Olive oil is selling at high rates, and the advance seems likely to continue. Cod-liver oil is quiet and unchanged. Cocaine has an upward tendency in value. The high price of opium is well maintained. Resorcin is almost unobtainable. Strychnine is dearer.

